# Flexible ureteroscopy training for surgeons using isolated porcine kidneys in vitro

**DOI:** 10.1186/s12894-015-0067-9

**Published:** 2015-07-23

**Authors:** Dongliang Hu, Tongzu Liu, Xinghuan Wang

**Affiliations:** Department of Urology, Zhongnan Hospital of Wuhan University, Wuhan, China

**Keywords:** Ureteroscopes, Training, Porcine kidney, Teaching

## Abstract

**Background:**

To evaluate the feasibility of flexible ureteroscopy training by using isolated porcine kidneys and ureters in vitro.

**Methods:**

Twenty young urologists were randomly divided into four groups. Overall performance was assessed based on a global rating scale, pass/fail rating, total time to complete task, learning curve, incidence of trauma, and perforations. The effect of training was determined by comparing their performance in baseline with that in the post-test.

**Results:**

After the training, average operation time significantly decreased from 18 ± 3.4 min to 11 ± 1.2 min (*P* < 0.05). The urologists exhibited a relatively stable performance level after the sixth operation. Significant differences were observed between pre-test and post-test with respect to the global rating scale and the pass/fail rating (*P* < 0.05). However, the incidence of mucosal trauma and perforations did not change significantly (*P* = 0.26 and 0.35, respectively).

**Conclusions:**

The isolated porcine kidneys are convenient and intuitive models for young urologists to practice flexible ureteroscopy on.

**Electronic supplementary material:**

The online version of this article (doi:10.1186/s12894-015-0067-9) contains supplementary material, which is available to authorized users.

## Background

Minimally invasive technologies, including flexible ureteroscopy, on kidney stones have developed rapidly. Flexible ureteroscopy is characterized by a relatively high safety and less injuries. It is widely used in urological diagnoses and treatments [1]; the indications are summarized in Table 1. The disadvantages of flexible ureteroscopy include high price, high repair cost, and low reusability, thereby limiting its application in clinical practice [2, 3]. Young urologists need a long learning curve, but they are given less operation opportunity. Thus, simulated training or actual practice is particularly crucial to them [4].

**Table 1:** The indications for flexible ureteroscopy

	Indications
Flexible Ureteroscopy	holmium: YAG laser lithotripsy for treatment
	diagnostic ureteroscopy
	patients with hematuria but CT and fluorescence in situ hybridization (FISH) negative result
	solitary kidney
	hard to percutaneous nephroscope lithotripsy
	kidney stone less than 2 cm in diameter
	etc.

Unlike laparoscopic technique, flexible ureteroscopy is characterized by a relatively narrow field and a small operating channel. Flexible ureteroscopy training focuses on finding and locating renal calyces instead of on operating skills. To enable young urologists to understand and master flexible ureteroscopy intuitively within a short period, we trained 20 young urologists by using porcine kidneys in vitro. All operations were performed via modular flexible ureteroscopy. We report our experience in this paper.

## Methods

### Experimental preparation

Four pairs of fresh porcine kidneys (including the ureter) were purchased from a slaughterhouse on the day of the experiment. To achieve smooth insertion of the ureteroscope access sheath, all specimens were soaked in warm water before training to thaw or avoid stiffness. The modular semi-disposable PolyScope system has a deflexion angle of 225 ° and a working channel diameter of 3.6 F [5, 6]. The rigid ureteroscope and guidewire were prepared to deal with a few difficult operations.

Researchers often choose residents or medical students as training subjects. To avoid wasting time on training students with basic skills, we trained 20 urologists who have 5 years to 8 years of experience and who are qualified attending physicians because flexible ureteroscopy typically requires rigid ureteroscopy and cystoscopy skills. All the participants had assisted in flexible ureteroscopy surgeries, but none had performed the operation as lead surgeon. The participants have not yet performed this operation independently.

The study was approved by the Ethical Committee of Zhongnan Hospital of Wuhan University and carried out in compliance with the Helsinki declaration. Participants signed an informed consent prior to enrolment in the study.

### Surgical methods

#### Brief steps

**Fig. 1:**
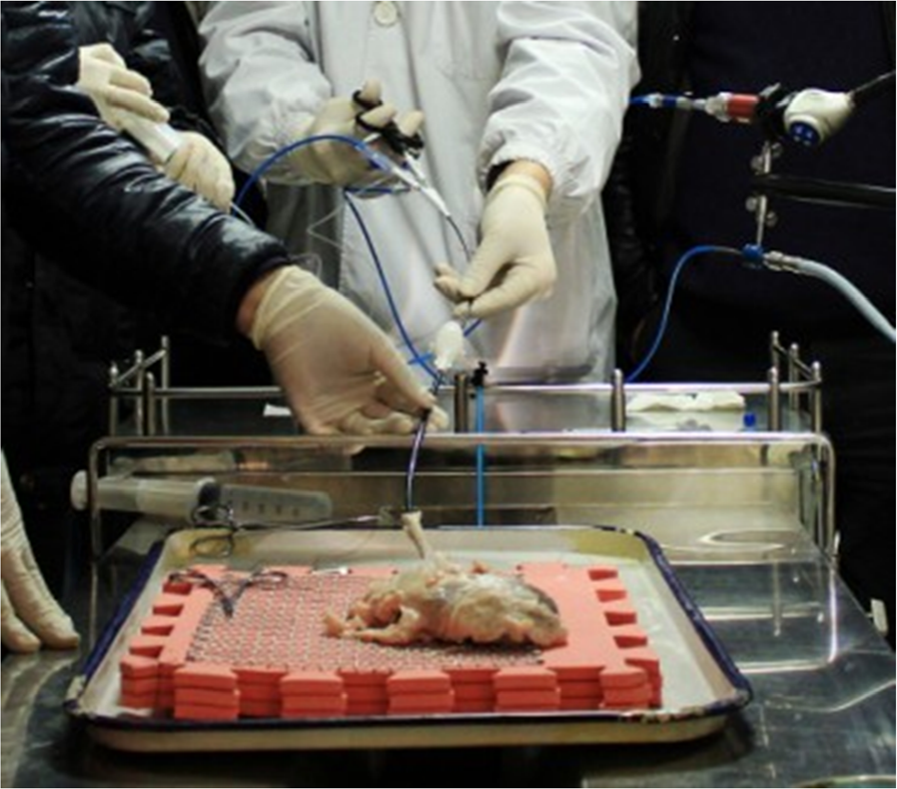
Isolated porcine kidney and ureter

The isolated porcine kidneys and ureters were placed and fixed on the net bar of the operating platform (Fig. 1). The ureteroscope access sheath was inserted into the ureteral orifice to establish a working channel for washing. The modular semi-disposable flexible ureteroscope was ready, and the focal distance was adjusted in vitro.

#### Pre-test preparation

A total of 20 young urologists were randomly divided into 4 groups. They attended a didactic teaching session given by a tutor, who provided a review of genitourinary anatomy, instruments, endoscopic techniques, and related knowledge. A supervised hands-on practice session was conducted. The urologists were allowed to practice repeatedly with the instruments to familiarize themselves with the task. During this session, the tutor provided instructive feedback.

#### Main assessments

A pre-test was administered to all the urologists to assess their baseline endoscopic ability before the study, and then, a post-test was given after the training intervention. The urologists were evaluated during the procedure by an experienced tutor by using a global rating scale that assesses performance parameters (see Additional file 1) [7]. Other data included total time to complete the task, incidence of mucosal trauma from the endoscope or instruments, and the number of perforations. An evaluation (pass/fail rating) was also conducted after the training program (see Additional file 1). In this program, operation time was defined as the time elapsed between access sheath insertion and task completion.

#### Training contents

The urologists were instructed to insert the ureteroscope access sheath and finish the required task. To compare operation time easily and uniformly, the tutor assigned the same training content to all participants in each operation: looking for the foreign body placed randomly in the kidneys and telling the specific renal calix. The tutor explained the key differences between flexible and rigid ureteroscopies, such as hand–eye coordination during examination, cooperation points in moving the ureteroscope, and handling strength in transforming the visual field. To save time, other skills that are identical or similar to rigid ureteroscopy were not emphasized during training. All the urologists observed the left and right kidneys separately (i.e., from easy to complicated) step by step. We trained a total of four operations, and thus, each participant was provided with eight operating opportunities. In the training, the tutor reviewed their shortcomings and time-consuming points after each operation. As a result, the urologists can improve their skills in the next operation based on their own understanding, thus indicating the efficiency of training.

To make the training impressive, intuitive, and clear, we enhanced light-source intensity and observed its shadow through a renal capsule. In this manner, the urologists can expediently achieve real-time positioning, and the training results can be verified after surgery through renal parenchyma incision.

### Statistical analyses

All results were expressed as mean ± SE. The data was analyzed for statistical significance by using *t*-test, chi-square, and Mann–Whitney *U* test (SPSS 15.0 software). The differences were considered significant at *P* < 0.05.

## Results

All the urologists finished the training contents according to the assignment. Five urologists from each group individually practiced the procedure by using four pairs of left and right isolated porcine kidneys; thus, a total of eight operation times for each urologist were recorded.

**Fig. 2:**
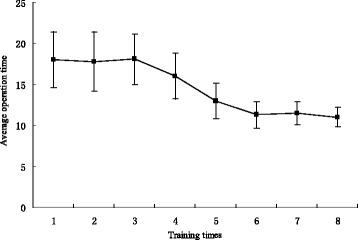
Learning curve of flexible ureteroscopy training

After the training, the average operation time significantly decreased from the initial 18 ± 3.4 min to 11 ± 1.2 min (*P* < 0.05). Significant differences were found between the pre-test and post-test with respect to the global rating scale and the pass/fail rating (*P* < 0.05, Table 2). However, the difference between the incidence of mucosal trauma and the number of perforations from the pre-test to post-test was insignificant (*P* = 0.26 and 0.35, respectively; Table 2). Based on the learning curve (Fig. 2), we found that the urologists achieved a relatively stable performance level after the sixth operation.

**Table 2:** Testing performance

Variable	Pre-test	Post-test	*P* value
Global rating scale	19.3 ± 0.8	28.7 ± 1.1	<0.05
Pass rating	6/20	19/20	<0.05
Time to complete task (min)	18 ± 3.4	11 ± 1.2	<0.05
Mucosal trauma (No.)	0.8 ± 0.5	0.6 ± 0.3	0.26
Number of perforations	0.23 ± 0.2	0.19 ± 0.1	0.35

## Discussion

Compared with laparoscopic training, reports on flexible ureteroscopy training are relatively rare. Urologists have been trained by using validated simulators or computer-based models [8, 9] without true anatomical experience and simulation environment [10]. Animal surgery has also been performed by using pigs under general endotracheal anesthesia [11]. By using pigs as models, the following factors should be considered before training. A double J tube must be placed on the pigs 2 weeks to 4 weeks before surgery to insert the ureteroscope access sheath that will be used as a working channel during surgery. This process is complex and time consuming with disregard to anesthesia or operating procedures. The key point of flexible ureteroscopy training is to observe the different renal calyces, particularly the lower ones, and thus, a live/intact pig or an enclosed abdomen is unnecessary. By contrast, carbon dioxide pneumoperitoneum is needed in laparoscopic training to perform the operation in a closed space. We conducted trainings by using pigs in the early stages, and found that inserting an ureteroscope access sheath and performing the subsequent operations are difficult because experimental pigs are relatively young. Unlike adult pigs, the urethra and ureter of young pigs are considerably thinner, and thus, the trainees cannot finish the training program easily.

Based on these situations, we used isolated porcine kidneys as training objects. Compared with a live/intact pig, the main advantages and characteristics of isolated porcine kidneys are summarized as follows. (1) Omission of the preoperative preparation, thereby increasing training and learning time. (2) As training objects, isolated kidneys are cheaper and easier to obtain than experimental pigs, thereby reducing training cost. (3) Changes in the visual field caused by different handling manipulations can be shown intuitively and clearly. The specific advantage of a modular flexible ureteroscope is its lens, which can only bend in one direction. However, the ureteroscope body and handle can be moved forward and backward and rotated 360°. In addition, the visual field of flexible ureteroscopy is different from that of rigid ureteroscopy, which has a linear scan imaging characteristic because unconscious shaking and handle grip strength change the direction of the lens and the visual range. All these differences emphasize the difficulty of flexible ureteroscopy training. To enable the urologists to master a sense of position as soon as possible, we enhanced the brightness of light to allow it to penetrate the surface of the kidney. Under such condition, verifying whether target renal calyces are reached accurately is convenient, and positioning differences between rigid and flexible ureteroscopy can then be compared visually. However, these experiences could not be accumulated in an experimental pig during endoscopic operation. (4) Side injury and complications of flexible ureteroscopy can be summarized intuitively. At the start of the preliminary training, the urologists inserted a flexible ureteroscope directly without placing an access sheath because of the relatively low temperature and stiff ureter. As a result, renal subcapsular edema and swelling occurred during water irrigation several minutes later. Afterward, the sheath could be inserted smoothly when the isolated kidneys and ureters have been soaked in warm water for a certain period of time. This process cautions the young urologists to learn from the training and avoid side injury during actual clinical work because we could not find any kidney and perirenal tissue damage in vivo. (5) Various training programs are easy to increase. At the end of the training, we placed small stones or mung beans in the renal pelvis through the access sheath to simulate a realistic experience. The foreign bodies were washed with water randomly into one of the renal calyx, and then the trainees were instructed to find the foreign bodies accurately by using a flexible ureteroscope. After several trainings, the urologists gradually accumulated operating experience.

After the training, the ability of the urologists was significantly improved. The global rating scale and the pass/fail rating verified their advancement. In addition, with the improvement in proficiency, the time to finish the task was decreased gradually and a relatively stable level was achieved after several operations. Thus, for urologists with endourological experiences, mastering this technique is possible, primarily through animal training, as long as they mastered the differences and features of the two types of ureteroscopes, particularly eye–hand–ureteroscope coordination. In the subsequent study, we decided to train the 20 urologists during actual operations with patients to verify their performance. Moreover, the differences in side injuries, such as trauma and perforation, between the pre-test and the post-test were insignificantly different. We thought that these complications could be easily experienced by junior residents. Our subjects had endourological experience on rigid ureteroscopy and cystoscopy. Thus, with regard to flexible ureteroscopy training, the main differences are the visual field and the manipulation habit rather than the basic skills required in rigid ureteroscopy. Surgical errors/injuries are unrelated to technical skills, but to other skills, such as communication, teamwork, supervision, and decision making [12]. Different medical environments may also affect the occurrence of injuries.

## Conclusions

To flexible ureteroscopy, young urologists need a long learning curve, but they are given less operation opportunity. The isolated porcine kidneys are convenient and intuitive models for young urologists to practice flexible ureteroscopy on.

## Additional file

Additional file 1: 
**Global rating scale and pass rating.** It was used to assess trainees’ performance or proficiency during this surgical training.
