# Supplementary material for: Flexible ureteroscopy training for surgeons using isolated porcine kidneys in vitro
Source: BMC Urol. 2015 Jul 23;15:71. doi: 10.1186/s12894-015-0067-9 (PMC4512109; doi:10.1186/s12894-015-0067-9)
Supplement: Additional file 1: — Global rating scale and pass rating. It was used to assess trainees’ performance or proficiency during this surgical training. [file 12894_2015_67_MOESM1_ESM.pdf]

Global rating scale

Please circle the number corresponding to the candidate's performance in each category, irrespective of training level.

Respect for tissue	1<br>Scope frequently pushed into urothelial wall. Used unnecessary force with guidewire and/or basket	2	3<br>Scope occasionally pushed into urothelial wall. Careful handling of guidewire and/or basket for the most part	4	5<br>No trauma to urothelial wall with scope. Consistent and careful handling of guidewire and/or basket
Time and motion	1<br>Many unnecessary moves	2	3<br>Made some unnecessary moves but time more efficient	4	5<br>No unnecessary moves and time is maximized
Instrument handling	1<br>Needed to repeatedly attempt guidewire insertion and/or basketing of stone	2	3<br>Able to insert guidewire and basket stone within first few tries. Occasional awkward maneuver	4	5<br>Able to insert guidewire and basket with fluid motion and no awkwardness
Handling of endoscope	1<br>Frequently had scope pointing away from the center of the urethra or ureter. Scope poorly aligned during procedure	2	3<br>Had scope centered for the most part. Guidewire in view for the most part. Better use of scope angle during procedure	4	5<br>Scope always centered and guidewire always in view. Scope always set at a good angle throughout procedure
Flow of procedure and forward planning	1<br>Frequently stopped or need advice or assistance from examiner	2	3<br>Demonstrated the ability to think forward with relatively steady progression of procedure	4	5<br>Obviously planned procedure from beginning to end with fluid motion
Use of assistants	1<br>Failed to have assistants help with guidewire insertion and/or stone basketing	2	3<br>Appropriate use of assistants most of the time	4	5<br>Strategically used assistants to the best advantage at all times
Knowledge of procedure	1<br>Deficient knowledge. Needed specific instruction at most operative steps	2	3<br>Knew all important aspects of operation	4	5<br>Demonstrated familiarity with all aspects of operation

Pass rating

Would you feel confident in allowing this trainee to perform this procedure in the operating room?
YES NO
